# Bridging immunization and oral health: a role for paediatric dentists in mitigating vaccine hesitancy in Africa

**DOI:** 10.3389/froh.2026.1751729

**Published:** 2026-03-05

**Authors:** Taofeek Olalekan Ligali, Ahmed Bhayat, Maha El Tantawi, Moréniké Oluwátóyìn Foláyan

**Affiliations:** 1 Department of Preventive Dentistry and Child Dental Health, Faculty of Dentistry, College of Medical Sciences, University of Maiduguri, Maiduguri, Nigeria; 2 AFRONE Network, Faculty of Dentistry, Alexandria University, Alexandria, Egypt; 3 Department of Community Dentistry, University of Pretoria, Pretoria, South Africa; 4 Early Childhood Caries Advocacy Group, University of Manitoba, Winnipeg, Canada; 5 Oral Health Initiative, Nigerian Institute of Medical Research, Lagos, Nigeria; 6 Department of Pediatric Dentistry and Dental Public Health, Faculty of Dentistry, Alexandria University, Alexandria, Egypt; 7 Faculty of Dental Medicine, Alamein International University, New Alamein, Egypt; 8 Department of Child Dental Health, Obafemi Awolowo University, Ile-Ife, Nigeria

**Keywords:** health communication, HPV vaccination, immunization (vaccination), interprofessional collaboration, oral manifestations, public health, vaccine hesitancy

## Introduction

Vaccines represent one of the most effective public health interventions for preserving life and controlling infectious diseases (1). Yet, these hard-won gains are increasingly threatened by global vaccine hesitancy (2). Concerns often stem from fears of adverse biological interactions or questions about the necessity of vaccines for treatable conditions like malaria (3). In the wake of the COVID-19 pandemic, doubts about vaccine safety and efficacy have intensified worldwide, posing a critical threat—particularly to regions with fragile healthcare systems such as Africa (4, 5).

At its core, much vaccine hesitancy arises from a misunderstanding of normal immunological processes. Vaccines work by simulating an infection, triggering a controlled systemic inflammatory response essential for building protective immunity. On rare occasions, this predictable response may produce transient, benign oral symptoms like non-specific ulcers or mild mucosal reactions (3). Without proper context, however, these self-limiting signs are easily misconstrued by the public as dangerous adverse effects, fueling unnecessary anxiety and distrust.

In Africa, this dynamic is further complicated by a deeper tapestry of historical memory, sociocultural beliefs, and systemic realities. The 2003–2004 polio vaccine boycott in Northern Nigeria, for instance, was not simply an act of refusal but a response to legitimate concerns about trust, governance, and historical exploitation following allegations that vaccines were contaminated with sterilising agents (6, 7). That event led to the resurgence of polio in the country, illustrating how vaccine hesitancy operates at the intersection of geopolitics, religion, and public health (8). Similarly, distrust of Western medicine in parts of Africa is often rooted in memories of colonial medical experimentation and persistent healthcare inequities (9). Such misinterpretations underscore a failure in proactive health communication. When providers do not preemptively educate patients about the possibility of benign, transient reactions, an information vacuum is created; one rapidly filled by misinformation that conflates temporally associated but unrelated oral ailments with vaccination itself (3). This cascade of misunderstanding directly contributes to vaccination opt-outs, eroding community immunity, and threatening public health. This commentary aims to reframe the narrative by providing a scientific basis for rare oral manifestations and equipping paediatric dentists, often trusted frontline providers, with the knowledge and tools to address concerns, correct misinformation, promote vaccine confidence, and establish a strategic communication framework.

### The immunological intersection: oral cavity as a reflection of systemic response

Vaccines initiate a controlled, systemic immunoinflammatory cascade essential for building protective memory, and the oral cavity, given its unique microbiome and role as a major portal for pathogen entry, is intrinsically linked to this systemic immune activity (10, 11). Although antigen administration is a safe, decades-old practice endorsed by organisations such as the World Health Organization, it can occasionally provoke a pronounced, synergistic immune activation (12). It is within this heightened state of immunologic readiness that transient post-vaccination oral manifestations may emerge, not as adverse events but as benign indicators of a competent immune response.

One such manifestation is reactive lymphadenopathy, a classic and direct immune reaction that may present as swelling of the submandibular or cervical lymph nodes. Dental professionals should therefore include recent vaccination history—typically within one to two weeks—in the differential diagnosis of acute facial swelling to avoid misinterpreting this normal immune response as an odontogenic infection (13). Beyond lymph node involvement, the systemic inflammatory reaction can predispose individuals to transient aphthous ulcers or generalised mucositis, as immune stress is a known trigger for such disturbances (14). In rare instances, dysgeusia, often described as a metallic taste, may occur, likely due to cytokine-mediated effects on taste bud regulation (15). Importantly, these symptoms are self-limiting and typically resolve within 72–96 h.

In addition, the Th1-driven inflammatory response can temporarily exacerbate quiescent oral conditions, including recurrent herpes labialis and oral lichen planus (16). Similarly, patients with pre-existing gingivitis may experience a transient increase in bleeding or erythema, reflecting the amplification of underlying inflammatory pathways (17). These phenomena underscore the importance of understanding post-vaccination oral changes as physiological, self-resolving responses rather than complications.

### The paediatric dentist’s unique position

The paediatric dentist occupies a distinctive and multifaceted position in the healthcare landscape. Unlike physicians who administer vaccines and may be perceived as having a vested interest, dentists benefit from a position of perceived neutrality on the topic of vaccination. Their credibility is rooted in specialized expertise in the orofacial region, uniquely qualifying them to authoritatively discuss post-vaccination phenomena such as lymphadenopathy, mucosal ulcers, and taste disturbances. This positions paediatric dentists as trusted allies in a coordinated health promotion strategy, transforming the clinical encounter into a safe venue for communication where concerns can be addressed on a one-on-one basis (18).

However, a significant limitation must be acknowledged: in many African contexts, routine dental attendance during early infancy, particularly during the critical 6-, 10-, and 14-week immunization visits, is not yet normative. Primary tooth eruption begins around six months of age, and parents typically seek dental care only when symptoms arise rather than for the preventive establishment of a dental home (19). Consequently, most children do not access paediatric dental services during these early immunization windows. This reality does not negate the potential role of paediatric dentists but instead necessitates a strategic reframing of how and where they engage in vaccine advocacy. Historical experiences have profoundly shaped vaccine perceptions across the continent. In South Africa, concerns about HIV/AIDS treatment denialism under Thabo Mbeki's administration created lasting scepticism toward government health messaging (20). In Cameroon and Tanzania, rumours that tetanus vaccines contained birth control agents led to reduced uptake (21). These events are not distant memories but remain active reference points in community conversations about new vaccines, including those for COVID-19 and HPV.

The growing movement to integrate oral health care within primary healthcare in Africa presents a timely opportunity for paediatric dentists to extend their reach beyond traditional clinic settings. By situating themselves within primary care environments, dentists can see children at scheduled immunization visits, creating repeated, opportunistic touchpoints for reinforcing vaccine-related information (22) and playing a more substantial role in promoting childhood vaccination (5). Documenting a child's vaccination status as a standard component of the paediatric dental assessment serves a dual purpose: it aids in the differential diagnosis of orofacial swellings and opens a non-confrontational dialogue about immunization, thereby normalizing the topic within the dental context.

Table 1 illustrates that the immunization schedule for children in Africa, based on the World Health Organization's Expanded Programme on Immunization (plus HPV) (23), has specific connections to oral health. The table highlights both direct and indirect implications of vaccinations for the oral cavity, ranging from the route of administration (as with oral vaccines) to the prevention of diseases that manifest with severe oral symptoms, such as measles and diphtheria, and the prevention of HPV-related oropharyngeal cancers. Notably, the administration of the HPV vaccine in adolescence (9–14 years) coincides with a period when dentists are actively providing orthodontic care, monitoring wisdom teeth development, and reinforcing lifelong oral health habits, including oral cancer prevention (24). Thus, the table offers paediatric dentists a practical framework for seamlessly integrating vaccine advocacy into routine clinical practice, positioning them as essential allies in the broader public health goal of achieving high vaccination coverage.

**Table 1:** Immunization schedule and oral health links for children in Africa.

Vaccine	Typical schedule (WHO EPI for Africa)	Link to oral health
BCG (Tuberculosis)	At birth	While BCG has no direct oral effects, it represents the child's first entry into the healthcare system. The dentist's inquiry about this vaccine can reinforce the importance of the'well-child approach and establish the dental home as part of the child's overall health monitoring from birth. A child's overall health status, influenced by diseases like TB, can affect oral health and healing capacity (57). The dentist's inquiry about this vaccine can open a dialogue about overall health.
Oral Polio Vaccine (OPV) and Inactivated Polio Vaccine	OPV: At birth, 6, 10, 14 weeks IPV: 14 weeks	**Administration Route**: The oral administration of OPV is a tangible example of how a vaccine directly interacts with the oral cavity and mucosal immune system; a concept dentists can explain (58).
Pentavalent Vaccine (Diphtheria, Tetanus, Pertussis, Hepatitis B, *Haemophilus influenzae* type b)	6, 10, 14 weeks	**Diphtheria:** Can cause a characteristic pseudo-membrane in the mouth and throat, leading to severe sore throat and swallowing difficulties (59). Preventing this protects the oral mucosa.
Pneumococcal Conjugate Vaccine	6, 10, 14 weeks	Prevents pneumococcal diseases, which can include otitis media. Oral health and ear infections are not directly linked, but an overall reduction in childhood illness supports consistent dental care.
Rotavirus Vaccine (Oral)	6, 10, 14 weeks	**Administration Route:** Like OPV, this is another orally administered vaccine. Dentists can use this to discuss the role of the oral cavity and gut mucosa as a first line of immune defense (60).
Measles-Rubella	1st Dose: 9 months 2nd Dose: 15–18 months	**Measles:** Measles infection often presents with Koplik's spots (small white spots) in the mouth before the body rash (61), and can cause severe oral ulcerations, making the threat of the disease concrete and preventable.
Tetanus Toxoid (e.g., in booster doses)	Booster doses are often given later in childhood.	**Tetanus**: Causes lockjaw (trismus), a severe spasm of the jaw muscles (62). Preventing tetanus directly ensures normal jaw function and oral intake, which is fundamental to oral and overall health.
Human Papillomavirus (HPV) Vaccine	2 doses, 6 months apart, for adolescents (typically 9–14 years)	**Direct Link**: This is the most significant oral health link. HPV is a leading cause of oropharyngeal cancers (63). Dentists are on the frontline of oral cancer screening. Promoting the HPV vaccine is a direct, powerful way to prevent these cancers, aligning perfectly with their scope of practice.
Yellow Fever Vaccine	9–12 months (in endemic areas)	Prevents a systemic viral illness. A dentist aware of a patient's travel history to endemic areas can reinforce the importance of this vaccine as part of protecting the child's overall health, which includes oral health.

### Leveraging immunization platforms for broader reach

Although the immunization schedule theoretically aligns with the timing for establishing a dental home and preventing conditions like early childhood caries (25, 26), this intersection remains unrealized for most African children in current practice. Table 1, therefore, serves a dual purpose: it outlines the content of vaccine advocacy conversations during dental encounters, and it identifies oral health linkages that non-dental providers, such as immunization nurses and community health workers, can deliver when trained and supported by paediatric dentists. Achieving the full impact of childhood vaccination, however, requires paediatric dentists to extend their advocacy beyond clinical walls. Scheduled immunization contacts have proven to be effective touchpoints for delivering integrated services, including maternal and child health, nutrition, early childhood development, and health emergency responses, because of their predictable timing, broad population reach, and repeated engagement with families who may otherwise have limited healthcare contact (27–29). By collaborating in joint community outreach programs and integrating oral health providers into existing community health worker networks, paediatric dentists can disseminate consistent vaccine messages alongside oral health education, effectively bypassing the barrier of low dental attendance.

Adapting delivery models to meet families where they are is equally critical. Engaging expectant and new mothers during antenatal or immediate postnatal visits establishes the dentist's role before the child's teeth erupt, normalizing early oral health engagement (30). For later childhood vaccines, such as measles at 9–15 months and HPV at 9–14 years, partnerships with early childhood development centres and schools offer additional avenues for advocacy. To position paediatric dentists effectively as vaccine advocates, it is essential to understand the African-specific dynamics shaping vaccine hesitancy. Trust in health interventions is often mediated through traditional authority structures, such as religious leaders, community elders, and traditional healers, who may wield greater influence than biomedical professionals (6). The 2003 polio crisis in Nigeria demonstrated that vaccine acceptance improved dramatically when traditional and religious leaders were adequately engaged (31). Paediatric dentists must therefore recognize that parental vaccine decisions are rarely individual but are embedded in community consensus-building processes.

Collaboration with local public health agencies is needed to develop culturally resonant and linguistically appropriate health education materials. Messages linking vaccines directly to oral health outcomes are particularly powerful: emphasizing the role of the HPV vaccine in preventing oropharyngeal cancers aligns with the dentist's scope of practice and offers a tangible, long-term benefit parents may not have considered (32). Similarly, highlighting the oral ulcerations caused by measles makes the abstract threat of the disease concrete and preventable by vaccination (33). By introducing oral manifestations of vaccine-preventable diseases into public health messaging, paediatric dentists can use the relatable lens of oral health to explain complex immunology. Unlike in high-income countries, where misinformation spreads primarily through digital platforms, African misinformation ecosystems are hybrid—combining oral tradition, religious sermons, community gossip, and increasingly, social media (34). Rumours about vaccines causing infertility, altering religious identity, or serving as Western population control mechanisms have deep historical roots and are perpetuated through trusted community channels (35, 36). The legacy of colonial medicine, often coercive and extractive, has created durable distrust that requires sustained, transparent engagement to overcome (37). Paediatric dentists, positioned as healthcare providers not directly associated with vaccine administration, can bridge this trust deficit by offering scientific facts and culturally resonant counter-narratives that address these specific concerns.

### Interprofessional collaboration as a unified strategy

Addressing the complex issue of vaccine hesitancy demands a unified strategy that moves beyond isolated efforts, making formal interprofessional collaboration among oral healthcare providers, paediatricians, and vaccination clinics essential (38). In the diverse African context, such collaboration must be pragmatically designed to function where digital infrastructure is limited. Low-tech solutions, such as standardised shared care cards held by parents that include both vaccination records and dental visit notes, or structured referral forms that travel with the patient, can prompt providers across settings to discuss vaccine status and ensure continuity of care. This integrated approach offers multiple benefits. It ensures that families receive a consistent, authoritative message across all clinical touchpoints, reinforcing scientific truth and building trust in an environment often fragmented by misinformation (12, 38–42). These formal pathways create a supportive system where providers can proactively identify and manage hesitancy, while also improving the handling of benign post-vaccination oral events. A dentist informed about common immune responses can immediately reassure a parent that transient symptoms like aphthous ulcers are normal, thereby preventing misattribution and bolstering vaccine confidence (3). Within this framework, paediatric dentists contribute to improving vaccine uptake, focusing on communication, counselling, and referral rather than the physical administration of vaccines unless local policies and training expand their scope. For Africa's often fragmented health systems, transforming oral health professionals into active agents of vaccine confidence is vital for safeguarding public health gains and fostering a more resilient, well-informed community (4, 5).

### Curricular implications: preparing paediatric dentists for vaccine advocacy roles

Paediatric dentists are not inherently equipped to assume vaccine advocacy roles, even when there is legislative authorisation and growing calls for their involvement (43–45). Dental education in Africa has traditionally emphasised the clinical management of oral diseases, with limited integration of population-level public health competencies (46). As a result, dental education curricula rarely systematically address vaccine science, the immunological basis of oral manifestations, or communication strategies for mitigating vaccine hesitancy. This educational gap poses a significant barrier to the consistent implementation of the proposed advocacy model. Structured curricular integration is therefore essential to establish vaccine advocacy as a standardised competency, enabling more equitable and effective contributions to immunisation coverage across the continent (47, 48).

Reforms are needed at both undergraduate and continuing professional development levels. In undergraduate training, dental schools across Africa should incorporate specific learning objectives into paediatric dentistry and oral medicine modules. These include the basic immunology of vaccination and the biological basis for post-vaccination oral manifestations, the expanded programme on immunisation schedule and its intersections with oral health as outlined in Table 1, communication frameworks such as motivational interviewing for engaging hesitant caregivers, and ethical principles that ensure respectful, non-coercive dialogue respecting parental autonomy (49, 50). Case-based learning, presenting scenarios such as post-vaccination lymphadenopathy or parental concerns about HPV vaccination during orthodontic consultations, can build both clinical reasoning and communication confidence beyond theoretical knowledge (51).

At the continuing professional development level, national dental associations and regulatory bodies in Africa should consider developing accredited, contextually adapted short courses on vaccine science and health communication that address culturally specific concerns. Delivered through hybrid models, these courses can reach practitioners in both urban and rural settings (52). Incentivising participation through continuing professional development credits and linking training to expanded scope-of-practice certifications could further enhance uptake, creating a cadre of formally recognised vaccine champions within communities.

### Acknowledging and addressing implementation barriers

Implementing this model requires acknowledging potential barriers at multiple levels, beginning with systemic challenges. Across Africa, the low rate of early preventive dental attendance remains a fundamental obstacle, rooted in structural determinants such as the limited geographic distribution of paediatric dentists, the concentration of services in urban tertiary centres, out-of-pocket payment models that discourage preventive visits, and health policies that have yet to prioritise the establishment of early dental homes (53–56). At the provider level, time constraints during dental appointments present a valid concern, though this can be addressed by integrating brief, structured screening questions about vaccination status into routine medical history-taking and by equipping clinicians with efficient, pre-prepared talking points. Equally important is the training and incentivisation of dental professionals through continuing education programmes focused on vaccine science and communication techniques, with the potential offer of professional development credits to enhance engagement. At the parental level, some caregivers may initially question the dentist's role in vaccination discussions, a perception that can be preempted by framing conversations within the clear context of oral health. For instance, by explaining that assessing a child's oral health requires understanding their overall health, including protection against vaccine-preventable conditions such as oral ulcers and cancer. By proactively addressing these operational and perceptual challenges, the proposed strategy becomes more robust and feasible. However, overcoming structural barriers demands advocacy that extends beyond the clinic. Paediatric dentists and their professional associations must engage with ministries of health to integrate oral health into national immunization and child survival policies, advocate for the inclusion of early dental visits in essential health benefit packages, and support the deployment of dental therapists and oral health officers to primary health centres where immunization services are delivered. Without concurrent efforts to strengthen the oral health system itself, the proposed advocacy role will remain accessible only to the minority of children already engaged in dental care, thereby risking the exacerbation rather than reduction of health inequities.

### A phased implementation pathway

We recognise that the model proposed in this commentary is aspirational and will require deliberate structural reforms before it can be implemented at scale. A phased approach offers a pragmatic pathway forward. In the short term, paediatric dentists can focus on later childhood touchpoints, such as measles vaccination at 9–15 months and HPV vaccination at 9–14 years, where school attendance or routine dental visits provide more realistic opportunities for engagement. During these encounters, dentists can begin documenting vaccination status, offering reassurance about benign oral manifestations, and promoting HPV vaccination as an oral cancer prevention strategy. In the medium term, establishing collaborative outreach models is feasible, whereby paediatric dentists or supervised dental therapists conduct periodic sessions at high-volume immunization clinics. Such initiatives build visibility, demonstrate value, and generate data on effectiveness to support policy advocacy. In the long term, systemic integration must be pursued, including the training of community health workers in oral health and vaccine messaging, the inclusion of oral health indicators in immunization surveillance, and the progressive realisation of universal health coverage that encompasses early preventive dental care.

## Conclusion

Paediatric dentists, with their specific expertise, perceived neutrality, and inherent trustworthiness, represent an underutilised public health asset. Realising this potential, however, requires a clear-eyed assessment of current attendance patterns and a willingness to adapt the model to African realities. Although few infants currently access dental care during early immunisation windows, paediatric dentists can still contribute meaningfully by maximising the impact of every existing dental encounter, extending their reach through outreach and task-shifting, and advocating for systemic reforms that will enable future generations to benefit from integrated oral health-immunisation services. Strengthening this arm of the healthcare team is a vital and cost-effective public health strategy for achieving the equitable vaccine coverage envisioned in global health agendas. By pursuing a phased, context-appropriate approach, one that meets families where they are while working to expand where dental care can be delivered, paediatric dentists can help address vaccine hesitancy even as they work toward the longer-term goal of universal early dental homes.
